# Diarrhea Case Surveillance in the Enterics for Global Health *Shigella* Surveillance Study: Epidemiologic Methods

**DOI:** 10.1093/ofid/ofad664

**Published:** 2024-03-25

**Authors:** Hannah E Atlas, Bakary Conteh, Md Taufiqul Islam, Khuzwayo C Jere, Richard Omore, Doh Sanogo, Francesca Schiaffino, Mohammad Tahir Yousafzai, Naveed Ahmed, Alex O Awuor, Henry Badji, Jennifer Cornick, Erika Feutz, Sean R Galagan, Fadima C Haidara, Bri’Anna Horne, Md Ismail Hossen, Aneeta Hotwani, Eric R Houpt, Abdoulie F Jallow, Mehrab Karim, Adama Mamby Keita, Youssouf Keita, Farhana Khanam, Jie Liu, Thandizo Malemia, Alhagie Manneh, Christine J McGrath, Dilruba Nasrin, Maureen Ndalama, John Benjamin Ochieng, Billy Ogwel, Maribel Paredes Olortegui, Loyda Fiorella Zegarra Paredes, Tackeshy Pinedo Vasquez, James A Platts-Mills, Syed Qudrat-E-Khuda, Sonia Qureshi, Md Nazmul Hasan Rajib, Elizabeth T Rogawski McQuade, Shazia Sultana, Sharon M Tennant, Kirkby D Tickell, Desiree Witte, Pablo Peñataro Yori, Nigel A Cunliffe, M Jahangir Hossain, Margaret N Kosek, Karen L Kotloff, Firdausi Qadri, Farah Naz Qamar, Milagritos D Tapia, Patricia B Pavlinac

**Affiliations:** Department of Global Health, University of Washington, Seattle, Washington, USA; Medical Research Council Unit The Gambia, London School of Hygiene and Tropical Medicine, Fajara, The Gambia; Infectious Diseases Division, International Centre for Diarrhoeal Disease Research, Bangladesh, Dhaka, Bangladesh; Malawi Liverpool Wellcome Programme, Blantyre, Malawi; Department of Clinical Infection, Microbiology and Immunology, Institute of Infection, Veterinary and Ecological Sciences, University of Liverpool, Liverpool, United Kingdom; Department of Medical Laboratory Sciences, Kamuzu University of Health Sciences, School of Life Sciences and Health Professions, Blantyre, Malawi; Centre for Global Health Research, Kenya Medical Research Institute, Kisumu, Kenya; Centre pour le Développement des Vaccins du Mali, Bamako, Mali; Faculty of Veterinary Medicine, Universidad Peruana Cayetano Heredia, Lima, Peru; Division of Infectious Diseases and International Health University of Virginia, School of Medicine, Charlottesville, Virginia, USA; Department of Pediatrics and Child Health, The Aga Khan University, Karachi, Pakistan; Department of Pediatrics and Child Health, The Aga Khan University, Karachi, Pakistan; Centre for Global Health Research, Kenya Medical Research Institute, Kisumu, Kenya; Medical Research Council Unit The Gambia, London School of Hygiene and Tropical Medicine, Fajara, The Gambia; Malawi Liverpool Wellcome Programme, Blantyre, Malawi; Department of Clinical Infection, Microbiology and Immunology, Institute of Infection, Veterinary and Ecological Sciences, University of Liverpool, Liverpool, United Kingdom; Department of Global Health, University of Washington, Seattle, Washington, USA; Department of Global Health, University of Washington, Seattle, Washington, USA; Centre pour le Développement des Vaccins du Mali, Bamako, Mali; Center for Vaccine Development and Global Health, University of Maryland School of Medicine, Baltimore, Maryland, USA; Department of Medicine, University of Maryland School of Medicine, Baltimore, Maryland, USA; Infectious Diseases Division, International Centre for Diarrhoeal Disease Research, Bangladesh, Dhaka, Bangladesh; Department of Pediatrics and Child Health, The Aga Khan University, Karachi, Pakistan; Division of Infectious Diseases and International Health University of Virginia, School of Medicine, Charlottesville, Virginia, USA; Medical Research Council Unit The Gambia, London School of Hygiene and Tropical Medicine, Fajara, The Gambia; Medical Research Council Unit The Gambia, London School of Hygiene and Tropical Medicine, Fajara, The Gambia; Centre pour le Développement des Vaccins du Mali, Bamako, Mali; Centre pour le Développement des Vaccins du Mali, Bamako, Mali; Infectious Diseases Division, International Centre for Diarrhoeal Disease Research, Bangladesh, Dhaka, Bangladesh; School of Public Health, Qingdao University, Qingdao, China; Malawi Liverpool Wellcome Programme, Blantyre, Malawi; Medical Research Council Unit The Gambia, London School of Hygiene and Tropical Medicine, Fajara, The Gambia; Department of Global Health, University of Washington, Seattle, Washington, USA; Center for Vaccine Development and Global Health, University of Maryland School of Medicine, Baltimore, Maryland, USA; Malawi Liverpool Wellcome Programme, Blantyre, Malawi; Centre for Global Health Research, Kenya Medical Research Institute, Kisumu, Kenya; Centre for Global Health Research, Kenya Medical Research Institute, Kisumu, Kenya; Asociación Benéfica PRISMA, Iquitos, Loreto, Peru; Asociación Benéfica PRISMA, Iquitos, Loreto, Peru; Asociación Benéfica PRISMA, Iquitos, Loreto, Peru; Division of Infectious Diseases and International Health University of Virginia, School of Medicine, Charlottesville, Virginia, USA; Infectious Diseases Division, International Centre for Diarrhoeal Disease Research, Bangladesh, Dhaka, Bangladesh; Department of Pediatrics and Child Health, The Aga Khan University, Karachi, Pakistan; Infectious Diseases Division, International Centre for Diarrhoeal Disease Research, Bangladesh, Dhaka, Bangladesh; Department of Epidemiology, Emory University, Atlanta, Georgia, USA; Department of Pediatrics and Child Health, The Aga Khan University, Karachi, Pakistan; Center for Vaccine Development and Global Health, University of Maryland School of Medicine, Baltimore, Maryland, USA; Department of Medicine, University of Maryland School of Medicine, Baltimore, Maryland, USA; Department of Global Health, University of Washington, Seattle, Washington, USA; Malawi Liverpool Wellcome Programme, Blantyre, Malawi; Department of Clinical Infection, Microbiology and Immunology, Institute of Infection, Veterinary and Ecological Sciences, University of Liverpool, Liverpool, United Kingdom; Division of Infectious Diseases and International Health University of Virginia, School of Medicine, Charlottesville, Virginia, USA; Department of Clinical Infection, Microbiology and Immunology, Institute of Infection, Veterinary and Ecological Sciences, University of Liverpool, Liverpool, United Kingdom; Medical Research Council Unit The Gambia, London School of Hygiene and Tropical Medicine, Fajara, The Gambia; Division of Infectious Diseases and International Health University of Virginia, School of Medicine, Charlottesville, Virginia, USA; Center for Vaccine Development and Global Health, University of Maryland School of Medicine, Baltimore, Maryland, USA; Department of Medicine, University of Maryland School of Medicine, Baltimore, Maryland, USA; Department of Pediatrics, University of Maryland School of Medicine, Baltimore, Maryland, USA; Infectious Diseases Division, International Centre for Diarrhoeal Disease Research, Bangladesh, Dhaka, Bangladesh; Department of Pediatrics and Child Health, The Aga Khan University, Karachi, Pakistan; Center for Vaccine Development and Global Health, University of Maryland School of Medicine, Baltimore, Maryland, USA; Department of Medicine, University of Maryland School of Medicine, Baltimore, Maryland, USA; Department of Pediatrics, University of Maryland School of Medicine, Baltimore, Maryland, USA; Department of Global Health, University of Washington, Seattle, Washington, USA

**Keywords:** children, diarrhea, enterics, *Shigella*, vaccine

## Abstract

**Background:**

*Shigella* is a leading cause of acute watery diarrhea, dysentery, and diarrhea-attributed linear growth faltering, a precursor to stunting and lifelong morbidity. Several promising *Shigella* vaccines are in development and field efficacy trials will require a consortium of potential vaccine trial sites with up-to-date *Shigella* diarrhea incidence data.

**Methods:**

The Enterics for Global Health (EFGH) *Shigella* surveillance study will employ facility-based enrollment of diarrhea cases aged 6–35 months with 3 months of follow-up to establish incidence rates and document clinical, anthropometric, and financial consequences of *Shigella* diarrhea at 7 country sites (Mali, Kenya, The Gambia, Malawi, Bangladesh, Pakistan, and Peru). Over a 24-month period between 2022 and 2024, the EFGH study aims to enroll 9800 children (1400 per country site) between 6 and 35 months of age who present to local health facilities with diarrhea. *Shigella* species (spp.) will be identified and serotyped from rectal swabs by conventional microbiologic methods and quantitative polymerase chain reaction. *Shigella* spp. isolates will undergo serotyping and antimicrobial susceptibility testing. Incorporating population and healthcare utilization estimates from contemporaneous household sampling in the catchment areas of enrollment facilities, we will estimate *Shigella* diarrhea incidence rates.

**Conclusions:**

This multicountry surveillance network will provide key incidence data needed to design *Shigella* vaccine trials and strengthen readiness for potential trial implementation. Data collected in EFGH will inform policy makers about the relative importance of this vaccine-preventable disease, accelerating the time to vaccine availability and uptake among children in high-burden settings.

## Teaser

This article describes the epidemiologic methods used in the Enterics for Global Health *Shigella* surveillance study, a multicountry surveillance network that aims to establish current *Shigella* diarrhea incidence and consequence estimates and prepare potential clinical trial sites for a phase 3 vaccine trial.

Diarrheal diseases are a leading cause of morbidity and mortality in children <5 years of age with an estimated 500 000 deaths annually, ranking third in the global disability-adjusted life-year burden [1, 2]. The highest burden of diarrheal disease occurs in low- and middle-income countries (LMICs); approximately 20% of deaths among children aged 1–59 months in sub-Saharan Africa and South Asia are attributed to diarrhea [1]. *Shigella*, a gram-negative bacillus, is a leading cause of acute watery diarrhea and the leading cause of dysentery [3–7], responsible for an estimated 90 000 deaths each year in children <5 years of age [1]. Depending on the severity of diarrhea, the diagnostic test used, and the setting, *Shigella-*attributed acute diarrhea incidence rates can range from 0.4 to 105 cases per 100 child-years [3, 4, 6, 8].

Consequences of *Shigella* diarrhea can include intestinal protein loss [9–11], persistent and prolonged diarrhea [12, 13], elevated inflammatory markers associated with environmental enteric dysfunction [14], and, ultimately, linear growth faltering and stunting [15–22]. *Shigella-*associated linear growth faltering further accounts for an estimated 28% additional deaths beyond those directly attributed to *Shigella* and more than 2 million cases of moderate to severe stunting each year [23, 24]. In addition to the short- and long-term health consequences, *Shigella* diarrhea can cause significant financial burden on households and local healthcare systems due to the high cost of hospital admission, treatment and transport costs, and lost wages [25, 26] and is a leading cause of antibiotic use for diarrhea [27].

There are several promising *Shigella* vaccine candidates under development, including a quadrivalent biconjugate vaccine (Shigella 4V), which is in a phase 1/2 trial among adult and pediatric populations in Kenya [28, 29]. A field efficacy trial in the target population of children aged 6–35 months living in high-burden LMIC settings [30] will be necessary for regulatory approval and adoption of efficacious vaccines by country policy makers. Such trials will require a consortium of LMIC sites in settings with a documented high incidence of *Shigella*-attributed diarrhea (specifically of vaccine-preventable *Shigella* serotypes), a demonstrated track record of following Good Clinical Practice standards, high participant retention, and the laboratory capacity to confirm *Shigella* infection.

Establishing incidence rates of *Shigella* diarrhea traditionally requires a closed population, such as in a cohort study, with prospective capture of incident diarrheal episodes and fecal testing for *Shigella* spp. Cohort studies can be expensive and resource intensive. With frequent follow-ups, the disease of interest may be diagnosed and treated earlier than would be expected in a nonresearch setting, altering the disease spectrum toward a milder syndrome and underestimating the severity of the illness. Facility-based surveillance can overcome these limitations of traditional cohort studies. However, doing so requires estimating the size of the underlying population and accounting for children with the disease of interest who fail to present to surveillance facilities. Hybrid surveillance, involving triangulation of facility-based surveillance with population enumeration and healthcare utilization surveys (HUSs) nested within the catchment population of the selected healthcare facility, is an efficient study design for generating credible population-based incidence estimates as demonstrated from other studies [31–33]. Hybrid surveillance has been successfully used to estimate incidence of etiology-specific diarrhea [7, 31] and enteric fever [34–37] in several LMIC settings.

Utilizing a hybrid surveillance approach, the Enterics for Global Health (EFGH) *Shigella* surveillance study will estimate serotype- and disease severity-specific incidence rates of *Shigella* diarrhea and document the health and economic consequences of *Shigella* diarrhea in 7 country sites in Africa, Asia, and South America. Through this multicountry network and associated clinical and laboratory harmonization, selected EFGH sites will be ready to quickly implement rigorous and efficient vaccine trials and contribute critical data to policy makers about the relative importance of this vaccine-preventable disease, thereby accelerating the time to vaccine availability and uptake among children in high-burden settings.

## SPECIFIC AIMS

The primary aim of the EFGH study is to determine the incidence of *Shigella*-attributed medically attended diarrhea (MAD) in children aged 6–35 months in each of the EFGH country sites (Bangladesh, Kenya, Malawi, Mali, Pakistan, Peru, and The Gambia) and by serotype, severity, detection method (culture vs quantitative polymerase chain reaction [qPCR]), age, and season. Secondary aims include the following: to describe the prevalence of resistance to commonly used antibiotics in *Shigella* spp. isolates in each EFGH country site; to determine the risk of death, hospitalization, persistent diarrhea, diarrhea recurrence, and linear growth faltering associated with an episode of *Shigella* diarrhea; and to compare various severity definitions in their ability to distinguish *Shigella* from non-*Shigella* attributable diarrhea and predict risk of death or hospitalization in the subsequent 3 months. Additional aims quantifying cost and answering laboratory-specific questions are described elsewhere in this supplement [38–40].

## METHODS

### Study Design

The EFGH study will use a hybrid surveillance design involving facility-based enrollment of diarrhea cases (diarrhea case surveillance) with 3 months of prospective follow-up. *Shigella* spp. will be identified by microbiologic culture and qPCR testing in rectal swabs collected from children at enrollment [39, 40]. Cross-sectional population enumeration and HUSs with random community-based sampling will be used to estimate the number of children living in facility catchment areas and the healthcare-seeking pattern of children with diarrhea to calculate *Shigella* diarrhea incidence rates [41].

### Diarrhea Case Surveillance Procedures

#### Study Population

Children aged 6–35 months presenting to selected EFGH study health facilities (Table 1) with diarrhea who are confirmed to reside in the study catchment area, and whose primary caregiver reported the child planned to remain in their current residence for at least the subsequent 4 months, will be eligible for enrollment (Table 2).

**Table 1.** Enterics for Global Health *Shigella* Surveillance Study Recruitment Centers and Regional Partners

Country Site	Catchment Area	Partners	Recruitment Facilities (Study Working Hours)
Bangladesh	Dhaka	icddr,b	icddr,b Dhaka Hospital; EFGH Field Office; Dhaka Medical College and Hospital; Mugda Medical College and Hospital; Sir Salimullah Medical College and Hospital (Sunday–Thursday: 8:30 Am–5 Pm)
Kenya	Gem, Karemo, and Asembo, Siaya County	KEMRI; UMB	Siaya County Referral Hospital; Lwak Mission Hospital; Akala Health Center; Ndienya Health Center; Bar Agulu Health Center; Ongielo Health Centre (Monday–Friday: 8 Am–4 Pm)
Malawi	Blantyre City	Malawi -Liverpool Wellcome Research Programme; University of Liverpool	Ndirande Health Centre (Monday–Friday: 8 Am–4 Pm)
Mali	Bamako	Centre pour le Développement des Vaccins du Mali; UMB	Banconi CSCOM; Asacodjeneka CSCOM; Asacodjip CSCOM; CSREF Commune 1 (Monday–Friday: 8 Am–4 Pm)
Pakistan	Karachi	The Aga Khan University	Ali Akbar Shah VPT Center; Khidmat e Alam Medical Centre; Bhains Colony VPT Center; Abbasi Shaheed Hospital; Sindh Government Hospital; Karongi; Sindh Government Hospital; Ibrahim Hyderi (Monday–Saturday: 9 Am–3 Pm)
Peru	Iquitos, Maynas Province	Asociación Benéfica PRISMA; University of Virginia	Centro de Salud America; Centro de Salud San Juan Bautista; Centro de Salud Santo Tomas; Posta de Salud Progreso; Posta de Salud Modelo (Monday–Saturday: 8 Am–4 Pm)
The Gambia	Basse, Upper River Region	MRC Unit The Gambia; UMB	Basse Hospital and Gambisara Health Centre (Monday–Friday: 8 Am–4 Pm)

Abbreviations: CSCOM, Centres de Santé Communautaire; CSREF, Centres de Santé Référence; EFGH, Enterics for Global Health; icddr,b, International Centre for Diarrhoeal Disease Research, Bangladesh; KEMRI, Kenya Medical Research Institute; MRC, Medical Research Council; UMB, University of Maryland, Baltimore.

**Table 2.** Inclusion Criteria for Diarrhea Cases

Prescreening Eligibility	Screening Eligibility
1. Child is 6–35 mo based on records or caregiver reported age	1. Child is 6–35 mo of age as confirmed by date of birth
2. Child presented during site-specific working hours and is still present at time of interview	2. Primary caregiver and child plan to remain at their current residence for at least the next 4 mo
3. Diarrhea/dysentery/gastroenteritis is one of the complaints of the child	3. Primary caregiver is able to provide informed consent (legal age or emancipated minor) and provides consent within a common language for which translations are available
4. Child not currently enrolled or in active EFGH follow-up	4. Child presents to health facility with diarrhea (≥3 abnormally loose or watery stools in the previous 24 h) with or without the presence of blood
	5. Child resides within the predefined study area
	6. <4 h has passed since the child presented to a health facility
	7. Diarrhea episode is: • Acute (onset within 7 d of study enrollment) and • Represents a new episode (onset after at least 2 diarrhea-free days)
	8. Caregiver is willing to have child participate in follow-up visits at week 4 and month 3
	9. Willingness to have samples collected from the child (rectal swabs at enrollment)
	10. Site enrollment cap has not been met
	11. Child is not being referred to a non-EFGH facility at the time of screening

Abbreviation: EFGH, Enterics for Global Health.

#### Screening

Screening will occur in a 2-step process: first, to define the screened population and to capture information on children who present outside of working hours (pre-screening), and second, to identify eligible children (screening). All children appearing <5 years of age presenting to EFGH recruitment facilities with a complaint of diarrhea will be pre-screened using a standardized prescreening case report form (CRF). Those deemed eligible during pre-screening will be invited to undergo screening consent and, if obtained, move on to screening utilizing a standardized screening CRF. Presentations outside of working hours will be documented utilizing the prescreening CRF in a site-dependent manner, relying on facility registry abstraction or EFGH study-specific logs. Caregivers of children who are eligible after screening will undergo full informed consent. All English-language CRFs and informed consent forms can be found at ClinicalTrials.gov (NCT06047821). More children may be eligible than can be enrolled due to study resource constraints or higher than expected diarrhea presentations. In such situations, a site-specific enrollment cap and sampling strategy will be implemented as described in Table 3. Data from prescreening and screening will inform site-specific estimates of the proportion of children presenting with diarrhea to the EFGH health facility who are not enrolled in EFGH but are otherwise eligible, to derive adjusted incidence estimates.

**Table 3.** Enrollment Caps Were Implemented at Some EFGH Sites in Anticipation of the Number of Eligible Children Exceeding the 1400 Total Enrollment to Ensure Relatively Even Recruitment of Cases Over the Full 24 Month Period

Country Site^a^	Enrollment Cap Type	Enrollment Cap Description
Bangladesh	Weekly	14 participants per week
Kenya	Weekly	18 participants per week
Malawi	Daily	4 participants per day on Monday & Tuesday; 3 participants per day Wednesday through Friday
The Gambia	Monthly	Varied according to high/low diarrhea season
Pakistan	Fortnightly	35 participants through Dec 2023, 21 participants January 2024 through end of study

^a^No enrollment cap implemented at the Peru and Mali sites.

#### Informed Consent Process

Informed consent will be obtained from the accompanying caregiver, including an additional provision for the use of de-identified participant data and samples for future studies. Consenting materials will be developed in English and translated to French (Mali site), Spanish (Peru site), Dholuo (Kenya site), Bangla (Bangladesh site), Urdu (Pakistan site), and Chichewa (Malawi site) and administered in the caregiver's language of choice. The signed consent forms will be provided to the caregiver and a copy securely stored in the participant's study file. The informed consent process will follow local institutional and ethical review board standards, including site-specific definitions of caregiver eligibility for consenting on behalf of the child.

#### Enrollment of Diarrhea Cases

A full medical history, including information about the severity of the current diarrhea episode and comorbidities, will be ascertained through a structured caregiver interview. A detailed physical examination will be conducted by a trained EFGH study member and will include vital sign collection (temperature collected using Exergen TAT-2000 Temporal Artery Professional Thermometer [Exergen Corporation; Watertown, Massachusetts], heart rate, and respiratory rate), World Health Organization (WHO) dehydration assessment [42], and documentation of other signs and symptoms. Where possible, the physical examination will occur as part of acute management. Immunization history will be abstracted from the child's medical record if available, or ascertained by caregiver recall. Breastfeeding history; household water, sanitation, and hygiene conditions and behaviors; and caregiver education will be ascertained through caregiver interview. A standardized country-specific asset index questionnaire [43] will be administered at enrollment and used to construct wealth quintiles. Human immunodeficiency virus (HIV) information, including biological mother status, child HIV exposure and status, and HIV treatment and testing history, will be collected at the Malawi, Kenya, and Mali sites.

#### Medical Management

The EFGH study teams will integrate with the existing health facility staff to participate in clinical management of the child, with treatment of the sick child taking precedent over study enrollment procedures. Clinical management standards outlined in the 2013 WHO Pocket Book for Hospital Care of Children [44], WHO 2014 Integrated Management for Childhood Illness (IMCI) guidelines [42], and the 2022 WHO Essential Medicines Antibiotic Book [45] are encouraged (Table 4). National or facility-specific guidelines will supersede WHO guidelines if available. All clinical management decisions and treatments administered during the health facility visit and prescribed for the discharge period will be recorded in standardized questionnaires. In cases where EFGH study staff are not solely responsible for the clinical management of EFGH enrolled children, variation from the WHO or national/facility-specific guidelines will be documented. The study will provide basic backup medications and supplies required by treatment standards outlined in the WHO guidelines in the event of a facility or national stockout. Quarterly clinical guideline adherence reports will be provided to each site reporting per-guideline proportions of children who received the guideline-specific treatment per indication, as well as antimicrobial resistance data from *Shigella* spp. isolates.

**Table 4.** World Health Organization (WHO) Diarrhea Management Guidelines for Children ≤5 Years^a^

**Dehydration Management (Without Severe Acute Malnutrition)**	**WHO-indicated Treatment**					
Severe (Plan C-Facility)	Start IV fluid immediately (preferably ringers Lactate Solution [100 ml/kg]) Reassess every 15–30 mins Give ORS (5 ml/kg/hr) as soon as the child can drink Re-classify dehydration after 6 h in infant & 3 h in child and continue with A, B, C plan	<12 m age—30 ml/kg^b^ in one hour followed by 70 ml/kg over 5 h 12 m to 5 y age—30 ml/kg^b^ in 30 min followed by 70 ml/kg over 2.5 h				
Some (Plan B-Facility)	Give recommended ORS in clinic over 4 h	Age^c^	<4 m	4–<12 m	12–<2 y	2 y– ≤ 5 y
	Re-classify dehydration after 4 h and continue with A, B, C plan	Weight	<6 kg	6–<10 kg	10–<12 kg	12–19 kg
			200–400 ml	400–700 ml	700–900 ml	900–1400 ml
None (Plan A-Home)	Increase food and fluid intake to prevent dehydration					
**Dehydration Management (With Severe Acute Malnutrition & No Shock)**	**WHO-indicated Treatment**					
Severe (Plan C-Facility)	Give ReSoMal^d^ (or half-strength standard low ORS with added potassium & glucose) 5 ml/kg every 30 min for the first 2 h 5–10 ml/kg per h for the next 4–10 for the next hours on alternate hours with F75					
Some (Plan B-Facility)	Same as above					
None (Plan A-Home)	Not applicable (because child admitted)					
**Zinc**	**WHO-indicated Treatment**					
All children	Zinc supplementation for 10–14 d ≤6 m 10 mg per day >6 m 20 mg per day					
**Antibiotics**	**WHO-indicated Treatment**					
Dysentery	Ciprofloxacin (15 mg/kg) twice daily for 3 d OR based on local sensitivity Azithromycin (10 mg/kg) given once daily for 4 d OR based on local sensitivity IV/IM ceftriaxone at 50–80 mg/kg per day for 3 d (if child is severely ill or as second line treatment)					
Suspected cholera (age ≥2 y + severe dehydration + cholera present in area)	Erythromycin (12 mg/kg) four times a day for 3 d Ciprofloxacin 10–20 mg/kg twice per day for 5 d Cotrimoxazole 4 mg/kg trimethoprim & 20 mg/kg sulfamethoxazole twice a day					

Abbreviation: F75, full formula name; IM, intramuscular; IV, intravenous; ORS, oral rehydration solution; ReSoMal, rehydration solution for malnutrition; WHO, World Health Organization.

^a^World Health Organization. Pocket book of hospital care for children: guidelines for the management of common illnesses with limited resources. Geneva, Switzerland. WHO 2013; World Health Organization. Chart Booklet: Integrated Management of Childhood Illness. Geneva, Switzerland. WHO 2014; World Health Organization. The WHO Essential Medicines List Antibiotic Book: improving antibiotic AWaReness. Geneva, Switzerland. WHO 2021.

^b^Repeat if the radial pulse is still very weak or not detectable.

^c^Use the child's age only when you do not know the weight. The approximate amount of ORS required (in ml) can also be calculated by multiplying the child's weight (in kg) by 75.

^d^If rehydration still required at 10 h, give starter F-75 instead of ReSoMal, at the same times.

#### Daily Record

Children who stay at the health facility overnight will be visited by study staff daily to ascertain changes to the child's condition, including diarrhea severity indicators, as well as to capture medical management decisions and discharge outcome.

#### Anthropometry

Length (<24 months of age) or height (≥24 months of age), weight, and mid-upper arm circumference (MUAC) will be collected for all participants at the enrollment visit and at each follow-up visit (week 4, month 3). Recumbent length and standing height will be measured to the nearest 0.1 cm using a wooden ShorrBoard® (Weigh and Measure, LLC, Olney, Maryland). Weight (Tanita HD-314 digital scale) and MUAC will be measured prior to, and after, the administration of rehydration fluids (if indicated). Post-rehydration weight and MUAC will be used as the final anthropometric values. Children <24 months of age or those unable to stand on their own will be weighed with their caregiver holding them (combined weight) and the caregiver's weight measured alone will be subtracted from the combined weight, with the difference considered the child's weight. Children ≥24 months of age or those who can stand on their own will stand on the scale alone to measure weight. MUAC will be measured using a 25-cm single-slotted insertion tape to the nearest 0.1 cm (Weigh and Measure, LLC). All anthropometric measurements will be measured twice (with the average of the 2 considered the final value) and repeated a third time if the 2 measurements differ by ≥0.3 cm for MUAC, ≥0.2 kg for weight, and ≥0.5 cm for length/height (in which case, the average of the 3 measurements will be considered the final value). Digital scales will be calibrated on a weekly basis in accordance with a standardized procedure. ShorrBoard® and MUAC tape calibration will be done on an annual basis by the local weights and measures department following local country site guidelines. Any defective equipment will be removed from use and replaced.

A minimum of 1 gold standard measurer will be identified at each country site. Every 6 months, site teams will conduct an anthropometry standardization test. The standardization test includes the gold standard measurer and the EFGH staff team member(s) involved in measuring children enrolled in EFGH. Each person will take 2 independent height/length and MUAC measurements on 6–10 healthy children (nonstudy participants). Each measurer's results will be compared to the gold standard measurer (inter-measurer difference) as well as the repeat measurements by the same measurer (intra-measurer differences). The standardization test results will identify supportive supervision needs and direct refresher training content. Refresher trainings will be conducted every 6 months following study launch and at minimum, 5% of all children will undergo remeasurement by the gold standard measurer for quality control purposes.

#### Rectal Swab Collection and Processing

Three rectal swabs will be collected from each child at the enrollment visit as close to presentation as possible and prior to administration of antibiotic therapy, if indicated. Participants will be positioned either laying on their side or stomach (on the caregiver's lap) and rectal swabs will be gently inserted into the child's rectum, fully rotated 3 times, and slowly removed. Staff will examine the swab to ensure there is visible fecal material and then place the swab into the respective tube. In the rare scenario in which the participant's caregiver refuses direct rectal swab collection and the participant passes whole stool during the enrollment visit, the 3 rectal swabs will be created from a whole stool sample.

The 3 rectal swabs will be collected using Nylon flocked swabs (Copan Diagnostics). The first swab (FLOQswab™) will be placed in a dry transport tube (2-mL screw top vial compatible with a bead beater) and stored at −80°C for eventual qPCR testing. The remaining 2 swabs will be placed directly in Cary-Blair transport media (swab 2) (FecalSwab, 4C028S) and modified buffered glycerol saline (mBGS) (swab 3) (FecalSwab, mBGS made locally) and cultured for *Shigella* spp. (Table 5). If <3 swabs are collected, collected swabs will be prioritized in the aforementioned order. After approximately 12 months of recruitment, the order of the swabs collected for Cary-Blair and mBGS swab will be reversed (dry swab, mBGS swab, Cary-Blair swab) in case swab collection order impacts the amount of fecal material on the swab.

**Table 5.** Summary of Specimen Collection and Collection Materials in the Enterics for Global Health Study

Study Time Point	Specimen Type	Purpose	Collection Materials	Collection Time Point
Year 1 and Year 2	Rectal swab 1	qPCR	Copan Diagnostics FLOQSwab	Enrollment
Year 1	Rectal swab 2	*Shigella* culture	Copan Diagnostics FecalSwab Cary-Blair Collection and Transport System	Enrollment
Year 1	Rectal swab 3	*Shigella* culture	Copan Diagnostics FLOQSwab and locally made modified buffered glycerol saline	Enrollment
Year 2	Rectal swab 2	*Shigella* culture	Copan Diagnostics FLOQSwab and locally made modified buffered glycerol saline	Enrollment
Year 2	Rectal swab 3	*Shigella* culture	Copan Diagnostics FecalSwab Cary-Blair Collection and Transport System	Enrollment
Year 1 and Year 2	Whole stool	Backup qPCR, whole stool culture substudy (The Gambia and Bangladesh sites only); inflammatory biomarker substudy^a^ (The Gambia, Malawi, Kenya, Peru, Pakistan, Bangladesh sites only)	Locally procured stool cups and spatulas	Enrollment
Year 1 and Year 2	Dried blood spot	Seroepidemiology of age- and site-specific preexisting immunity against *Shigella*	Whatman 903 protein saver card	Enrollment, week 4 follow-up

Abbreviation: qPCR, quantitative polymerase chain reaction.

^a^Myeloperoxidase, calprotectin, lipocalin-2, and hemoglobin.

Quantitative PCR, using the TaqMan Array Card system, will be customized to detect gene targets for >30 enteric pathogens, including *Shigella* spp. [39]. *Shigella* spp. will also be isolated, speciated/serotyped, and subject to antimicrobial susceptibility testing (AST) using standard microbiologic methods from rectal swabs 2 and 3 [40].

#### Whole Stool Collection and Processing

At all EFGH sites, a whole stool sample will be collected if a bowel movement takes place at any point during the enrollment visit prior to the child leaving the facility and stored at −80°C. At The Gambia, Kenya, Malawi, Pakistan, Bangladesh, and Peru sites, if a participant does not produce a whole stool sample during the enrollment visit, the study staff will train the caregiver to collect the first whole stool passed upon returning home in the 24-hour period following enrollment. Aliquots of the whole stool stored at −80°C will be used for backup qPCR testing and tested in an inflammatory biomarker substudy [46]. At The Gambia and Bangladesh sites, a portion of whole stool sample collected at the enrollment visit (not from home visits) will be swabbed and placed in Cary-Blair (Year 1 FecalSwab, Copan Diagnostics, 4C028S) (or mBGS, Year 2) and another portion weighed and stored at −80°C for culturing and qPCR, respectively, as part of a rectal swab–whole stool *Shigella* comparison substudy [40].

#### Dried Blood Spot Collection and Processing

Dried blood spots will be collected using a single heel/finger prick at the enrollment and week 4 follow-up visits (Table 5). Less than 0.5 mL of blood will be collected from all enrolled children for a dried blood spot on Whatman® 903 protein saver cards, which will be used for eventual descriptive seroepidemiology of preexisting immunity against *Shigella* and description of immune responses to infection [47]. Cards will be fully dried at room temperature for a minimum of 2 hours, then placed in a plastic bag and refrigerated at 4°C for long-term storage.

#### Diarrhea Diary

On the day of enrollment, caregivers will be provided a diarrhea diary that includes visual cues. The diarrhea diary will capture daily information about the diarrhea episode over the 14-day period following enrollment, including diarrhea severity indicators (number of loose or watery stools, vomiting episodes, visible blood in stool, temperature/hot to the touch) and medications. At the enrollment visit, staff will explain the goal of the diary card, how to assess the signs and symptoms, how to complete the diary card, and strategize ways to ensure the diary is completed each day. Caregivers will also be reminded to return the diarrhea diary at their scheduled week 4 study visit or through another retrieval method. Site-specific diarrhea diary contact and collection plans can be found in Table 6 and a copy of the diary on ClinicalTrials.gov (NCT06047821).

**Table 6.** Site-Specific Diarrhea Diary Contact and Collection Plans in the Enterics for Global Health Study

Country Site	Contact Strategy	Collection Strategy
Bangladesh	Phone calls every 4 days starting day 1	Returned by the caregiver at the week 4 follow-up visit
Kenya	Phone call (or SMS message if no answer) on day 3	Returned by the caregiver at the week 4 follow-up visit or after completion (day 15 onward)
Malawi	Home visit on day 1	Collected by study staff from the home within 3 day after completion (starting d 15)
Mali	Home visit on day 3	Collected by study staff from the home within 3 day after completion (starting day 15) Returned by the caregiver at the week 4 follow-up visit (with reminders 1 week and 1 day before the week 4 follow-up visit)
Pakistan	Home visits on day 3 and day 10 and phone call reminder on day 2 and day 7	Collected by study staff from the home on day 15 or 16 or returned by the caregiver at the week 4 follow-up visit
Peru	Phone call or home visit on day 3 and day 6	Collected by study staff from the home on day 15
The Gambia	Phone call on day 5 (±2 days), day 9 (±2 days), and day 15 (+2 days)	Returned by the caregiver at the week 4 follow-up visit

Abbreviation: SMS, short messaging service.

#### Follow-up of Culture-Positive *Shigella* Cases

*Shigella* culture and AST results will be communicated to clinical teams as soon as they are available (approximately 3–5 working days [maximum 7 days] after enrollment). If *Shigella* spp. are isolated, the participant's caregiver will be contacted by the study staff within 24 hours of culture results availability to determine the health status of the child. If the child has not improved or is deteriorating, the caregiver will be encouraged to return to an EFGH facility for further evaluation and possible treatment changes guided by AST results.

#### Follow-up

Scheduled study follow-up visits will take place 4 weeks (visit window: 24–37 days since enrollment) and 3 months (visit window: 84–97 days postenrollment) from the date of enrollment at an EFGH recruitment facility or the participant's home in settings where returning to health facilities is not feasible. If >30 days pass since the upper limit of the scheduled follow-up visit window (>67 days and >127 days post-enrollment for the week 4 visit and month 3 visits, respectively), the follow-up visit will be considered missed. A participant is considered lost to follow-up if they have missed both the week 4 and month 3 visits.

The participant's general condition will be assessed at the start of each follow-up visit. Using a standardized questionnaire, caregivers will be interviewed about the child's health history since the last study visit and the participant's recovery from the index diarrheal episode, new diarrheal episodes and associated care-seeking behavior, recent hospitalizations, and other illnesses. Information on the cost of the index diarrhea episode, including any medications obtained following the enrollment visit or other indirect costs incurred (transport, household costs, missed income-generative activities) will be documented [38]. Anthropometry (length/height, MUAC, and weight) will be collected. A dried blood spot will be collected at week 4 follow-up visit for seroepidemiology [47].

Unwell children who return to EFGH health facilities outside of regularly scheduled visits (such as due to illness) will complete an unwell child visit. During this visit, the participant's health status will be reassessed and documented by the study clinicians, and treatment and/or additional clinical observation will be provided if indicated.

#### Cause of Death Ascertainment

For participants who die, site teams will abstract available information from the death certificate. In the absence of a death certificate, the date of death will be collected based on either the participant's medical record (for inpatient deaths), caregiver report, or community knowledge. Study staff will determine whether the caregiver is willing to participate in a voluntary mortality interview. This interview aims to capture events and conditions surrounding the death and is an open-ended interview adapted from the 2016 WHO Verbal Autopsy Instrument [48] administered by a trained staff to the caregiver of the deceased child. If the death occurred in the health facility and no death certificate is available, an *International Classification of Diseases, 10th Revision* (*ICD-10*)–trained clinical staff will conduct a brief interview with the clinician who managed the participant. This interview will include documenting all events that lead to the participant's death. The interviewer will assign a preliminary cause of death based on the clinical staff interview and *ICD-10* training. The final cause of death will be determined by a mortality review panel of pediatric clinical experts who have trained on the WHO death certification guidance and the *International Classification of Diseases, 10th Revision* (*ICD-10*) [49]. This panel will be convened midpoint of the study and at study close. The panel will collate data from deceased participants’ medical records, death certificates, and caregiver interviews, and *ICD-10*–compliant causes of death will be determined by consensus.

### Study Tool Development

Standardized study tools, including the central protocol, CRFs, standard operating procedures (SOPs), study logs, and template consent forms were developed by representatives from across the consortium in working groups organized by domain: diarrhea case surveillance, population enumeration and the HUS, microbiology, and data management. Final CRFs, SOPs, and consent forms for study were translated into local languages and pilot tested with volunteers at each site. Piloted CRFs were scanned and securely shared with the central coordinating team for review, accompanied by written feedback. A structured debrief of piloting activities was conducted in the working group forum.

### Training and Standardization

To ensure high-quality data collection and standardization across sites, a 5-day training of trainers (ToT) workshop was held for the diarrhea case surveillance domain. The ToT model [50] was chosen due to efforts to limit gathering size and international travel during the SARS-CoV-2 pandemic and, importantly, to leverage consortium technical expertise, facilitate capacity building and knowledge sharing between site teams, and equip lead trainers with content expertise to conduct site-level trainings. The in-person training employed adult learning principles, didactic training methodologies, and integrated elements of Good Clinical Practice. Training curriculum included best practices of human subjects research, clinical management, specimen collection, anthropometry, and diarrhea case surveillance data collection and systems. A pre- and post-test was administered prior to the start and at the culmination of the training session to evaluate competency. Ahead of the ToT, participants completed an online training module that consisted of 9 pre-recorded video presentations providing a high-level overview of the EFGH study background and rationale and walked through procedures and other critical aspects of the study. Slide decks and Spanish- and French-translated transcriptions were also available. This online module will be available to all EFGH team members and is a requirement for any newly onboarded staff. A separate sitewide training, identical in content, was conducted at the Peru site due to challenges with travel logistics.

After ToTs, trainers developed individual site training plans. Site training activities, staff pre- and post-test comprehension scores, and training evaluations were documented in a standardized report template and reviewed centrally to ensure standardization across sites.

### Statistical Analysis

#### Analytic Methods

The statistical analysis plan for the EFGH study is being developed by a statistical working group comprised of representatives from site and coordination team members of the consortium and will be finalized prior to the end of diarrhea case surveillance enrollment (September 2024). The current version, and its subsequent updates, can be found on ClinicalTrials.gov (NCT06047821). Here, we summarize the statistical analysis methods of the incidence estimation.

The crude incidence of *Shigella* MAD will be calculated as the sum of total *Shigella* diarrhea cases (confirmed by either culture or qPCR) divided by the child-years at risk of children 6–35 months of age in the defined catchment area. Adjusted incidence rates of *Shigella* diarrhea will be calculated by the crude incidence divided by the proportion of otherwise eligible children who were not enrolled in EFGH and the proportion of children aged 6–35 months in the catchment area with diarrhea in the last 2 weeks who sought care at an EFGH facility. At a minimum, the care-seeking proportion will be estimated separately for watery diarrhea and dysentery because of known differences in care-seeking behavior [51]. Differential care-seeking by additional variables such as age, wealth quintile, and other severity characteristics including dehydration status may be accounted for depending on the numbers observed in the HUS and comparability with the enrolled diarrhea cases. Child-years at risk for *Shigella* diarrhea will be computed as the product of the estimated number of children 6–35 months of age in the catchment area enumerated during population enumeration activities and the facility-specific period of follow-up (approximately 24 months). Incidence rates will be presented for each diagnostic method (culture confirmed and molecularly confirmed).

*Shigella* diarrhea incidence will also be calculated by species (*S. flexneri, S. sonnei, S. dysenteriae, S. boydii*) and by *S. flexneri* serotype/subserotype [52] and by the combined vaccine-preventable serotypes. We will similarly stratify incidence by age at enrollment visit (6–11 months, 12–17 months, 18–23 months, or 24–35 months); by mild, moderate, and severe categories from various diarrhea severity definitions [42, 53–57] and by whether or not a child was hospitalized; by laboratory method (culture or qPCR); and by month to capture seasonality.

#### Sample Size Calculation for Diarrhea Cases

The minimum number of culture-confirmed *Shigella* cases (numerator) per site was set at 65 to ensure at least 5 isolates from the 4 leading species/subserotypes (*S. sonnei*, *S. flexneri* 2a, *S. flexneri* 3a, and *S. flexneri* 6) at each site and to achieve appropriate precision (width less <3%) for a 2-sided 95% confidence interval around the observed proportion of children enrolled with culture-positive *Shigella*. We assumed that 4.7% of diarrhea cases in EFGH would be *Shigella* spp. culture positive [58], requiring that we enroll 1400 participants presenting to health facilities with diarrhea over the 24-month period (∼58 participants per month).

### Ethical Considerations

Central versions of the EFGH study protocol, consent forms, CRFs, and other study documents were created and subsequently adapted to meet ethical- and institutional review board (IRB)–specific standards. Prior to study initiation, site-specific study materials were approved by the IRBs at each EFGH site and affiliated coordinating body. This study will be conducted according to Good Clinical Practice, including Good Clinical Laboratory Practice, the Declaration of Helsinki, and IRB and local rules and regulations specific to each EFGH country.

## STRENGTHS AND LIMITATIONS

EFGH will utilize standardized and rigorous field research and laboratory techniques to measure the incidence and consequences of *Shigella* in young children living in high-burden settings. With several Global Enteric Multicenter Study (GEMS) [3], GEMS-1A [59], and Vaccine Impact on Diarrhea in Africa (VIDA) [7] sites participating in EFGH and utilizing similar methods, data can provide a 15-year overview of the evolving incidence, etiology, and adverse clinical outcomes of MAD at these sites. However, EFGH has several limitations, including variability in clinical assessments, the dependence on caregiver recall to construct severity definitions, shortcomings of the incidence-rate adjustments versus direct incidence estimation, and the types of cases that facility-based surveillance detects.

A key aim of EFGH is to estimate the incidence of *Shigella* diarrhea by various severity definitions, which include clinical measures such as dehydration and clinician decision to hospitalize. While clinical assessments will be completed by clinical personnel trained in the EFGH clinical SOPs and trained in WHO IMCI guidelines, inter-observer reliability in the assessment of dehydration can be challenging [60], suggesting there may be over- or underestimation of dehydration within, and between, EFGH sites. Duration of diarrhea, fever, and vomiting are also key components of many severity definitions, and symptoms observed during more recent days will be more accurately recalled by caregivers than those temporally farther from the day of diarrhea presentation, with more severe disease more likely to be recalled [61].

A key assumption of the hybrid surveillance design is that enrolled cases are a representative sample of cases from the underlying population. Meeting this assumption requires that the recruiting EFGH health facilities are those that equitably serve the catchment area population. Care-seeking behavior, including whether or not to seek care and where to seek care, may be affected by perception of the illness, and less significant illnesses may not present to certain types of facilities. Other factors include socioeconomic status, age, and perceived quality of care. Care-seeking adjustments will also be limited by the number of children included in the HUSs, leading to imprecision in adjusted incidence estimates.

EFGH screening and enrollment is limited to working hours, which is more feasible from a cost, staffing, and logistic perspective than undertaking this activity 24 hours a day, 7 days a week. While every effort will be made to ascertain screening data from logs and pre-screening forms, it is possible that some cases are missed. Also, because children presenting outside of working hours will not undergo real-time screening, including a clinical history, as do those who present during working hours, EFGH will not be able to quantify whether severity was similar between those presenting during working hours and those presenting outside. Since presentation to health facilities for care on weekends or evenings is likely the result of major caregiver concern, either because the child is very sick or very young, it could be that those cases presenting outside of working hours have more severe disease or are younger, leading EFGH to underestimate the severity of *Shigella* diarrhea cases. The range of the extremes (assuming all cases presenting outside of working hours had severe diarrhea or that all had mild diarrhea) will be presented in sensitivity analyses to determine the potential impact of the assumption that cases presenting within working hours were representative of those presenting outside of hours.

Finally, EFGH is enrolling children presenting to health facilities with any severity of diarrhea, rather than a subset of children with more severe diarrhea, to enable an assessment of various definitions of moderate and/or severe presentations of *Shigella* diarrhea and to estimate the incidence of all medically attended *Shigella* diarrhea. However, this decision comes at the cost of potentially having less power to detect important consequences of *Shigella,* such as linear growth faltering, diarrhea recurrence, and/or death, all of which are more common in children with more severe diarrhea.

## CONCLUSIONS

Ensuring the success of eventual *Shigella* vaccine field efficacy trials and promoting uptake and adoption of newly licensed *Shigella* vaccines by policy makers will require recent, country-specific data on the burden of *Shigella* diarrhea as well as the health and economic consequences of this important disease. The EFGH consortium will provide updated estimates of the incidence and consequences of culture- and molecularly confirmed medically attended *Shigella* diarrhea in young children living in high-burden settings where *Shigella* vaccines are likely to have the greatest impact on child health.

## Notes

***Author contributions.*** H. E. A., B. c., M. T. I., K. C. J., R. O., D. S., F. S., M. T. Y., N. A. C., M. J. H., M. N. K., K. L. K., F. Q., F. N. Q., M. D. T., and P. B. P. actively participated in monthly working group meetings during which the conceptualization and outline was discussed and agreed-upon. H. E. A., B. c., M. T. I., K. C. J., R. O., D. S., F. S., and M. T. Y. (with review, scientific input, and editing from N. A. C., M. J. H., M. N. K., K. L. K., F. Q., F. N. Q., M. D. T., and P. B. P.) wrote the first draft of the manuscript. N. A., A. O. A., H. B., J. C., E. F., S. R. G., B. H., M. I. H., A. H., E. H., A. F. J., M. K., A. M. K., Y. K., F. K., J. L., T. M., A. M., C. J. M., D. N., M. N., J. B. O., B. O., M. P. O., L. F. Z., T. P. V., J. P. M., S. Q., M. N. H. R., E. T. R. M., S. S., S. T., K. D. T., D. W., and P. P. Y. reviewed and edited the manuscript. All authors approved the content of the final manuscript.

***Acknowledgments.*** We are very grateful for the partnerships that make this work possible, including ministries of health, health facilities, and community partners, all of whom have worked tirelessly to support planning for the implementation and eventual launch of the EFGH study. The EFGH study staff from each of the study sites and coordinating institutions have worked together diligently, mostly remotely due to COVID-19–related travel restrictions, to identify scientific aims, develop study procedures, and participate in training activities with mutual respect and absolute collaboration. We thank Anya Lewin for her project management support of this supplement. Most importantly, we would like to thank the eventual study participants and their families for taking part in this research study.

***Disclaimer.*** The views expressed are those of the author(s) and not necessarily those of the National Institute for Health and Care Research (NIHR), the Department of Health and Social Care, the UK government, or the UK Health Security Agency.

***Supplement sponsorship.*** This article appears as part of the supplement “Enterics for Global Health (EFGH) *Shigella* Surveillance Study-Rationale and Methods,” sponsored by the Bill & Melinda Gates Foundation.

***Financial support*.** This project is supported by the Bill & Melinda Gates Foundation (grant numbers INV-016650, INV-031791, INV-036891, INV-036892, INV-028721, INV-041730, INV-044311, and INV-044317) and the National Institutes of Health (grant numbers D43TW010913 to M. N. K. and M. P. O. and K43TW012298 to F. S.). The Gambia team's work is also supported by the United Kingdom Research and Innovation Medical Research Council (program number MC_UU_00031/1–Disease Control and Elimination). N. A. C. is an NIHR Senior Investigator (NIHR203756). N. A. C., J. C., and K. C. J. are affiliated with the NIHR Global Health Research Group on Gastrointestinal Infections at the University of Liverpool and the NIHR Health Protection Research Unit in Gastrointestinal Infections at the University of Liverpool, a partnership with the UK Health Security Agency in collaboration with the University of Warwick.
